# Using ChatGPT in Psychiatry to Design Script Concordance Tests in Undergraduate Medical Education: Mixed Methods Study

**DOI:** 10.2196/54067

**Published:** 2024-04-04

**Authors:** Alexandre Hudon, Barnabé Kiepura, Myriam Pelletier, Véronique Phan

**Affiliations:** 1 Department of Psychiatry and Addictology, University of Montreal, Montreal, QC, Canada; 2 Faculty of Medicine, Université Laval, Québec, QC, Canada; 3 Department of Pediatrics, Université de Montréal, Montreal, QC, Canada

**Keywords:** psychiatry, artificial intelligence, medical education, concordance scripts, machine learning, ChatGPT, evaluation, education, medical learners, learning, teaching, design, support, tool, validation, educational, accuracy, clinical questions, educators

## Abstract

**Background:**

Undergraduate medical studies represent a wide range of learning opportunities served in the form of various teaching-learning modalities for medical learners. A clinical scenario is frequently used as a modality, followed by multiple-choice and open-ended questions among other learning and teaching methods. As such, script concordance tests (SCTs) can be used to promote a higher level of clinical reasoning. Recent technological developments have made generative artificial intelligence (AI)–based systems such as ChatGPT (OpenAI) available to assist clinician-educators in creating instructional materials.

**Objective:**

The main objective of this project is to explore how SCTs generated by ChatGPT compared to SCTs produced by clinical experts on 3 major elements: the scenario (stem), clinical questions, and expert opinion.

**Methods:**

This mixed method study evaluated 3 ChatGPT-generated SCTs with 3 expert-created SCTs using a predefined framework. Clinician-educators as well as resident doctors in psychiatry involved in undergraduate medical education in Quebec, Canada, evaluated via a web-based survey the 6 SCTs on 3 criteria: the scenario, clinical questions, and expert opinion. They were also asked to describe the strengths and weaknesses of the SCTs.

**Results:**

A total of 102 respondents assessed the SCTs. There were no significant distinctions between the 2 types of SCTs concerning the scenario (*P*=.84), clinical questions (*P*=.99), and expert opinion (*P*=.07), as interpretated by the respondents. Indeed, respondents struggled to differentiate between ChatGPT- and expert-generated SCTs. ChatGPT showcased promise in expediting SCT design, aligning well with *Diagnostic and Statistical Manual of Mental Disorders, Fifth Edition* criteria, albeit with a tendency toward caricatured scenarios and simplistic content.

**Conclusions:**

This study is the first to concentrate on the design of SCTs supported by AI in a period where medicine is changing swiftly and where technologies generated from AI are expanding much faster. This study suggests that ChatGPT can be a valuable tool in creating educational materials, and further validation is essential to ensure educational efficacy and accuracy.

## Introduction

### Undergraduate Medical Education

Undergraduate medical studies offer a wide range of learning opportunities through various teaching methods for medical students [1]. The competencies required are partly dictated by the Medical Council of Canada, and these skills are regularly assessed throughout the undergraduate medical education (UGME) program. Training programs must incorporate clinical reasoning instruction to aid students in developing this crucial competency [2]. The Bloom taxonomy is a useful tool for clearly identifying the cognitive level targeted by different teaching methods [3]. The taxonomy helps determine the appropriate methods for teaching and evaluating students based on the desired level of competency. Although various teaching methods are used, clinical situations followed by multiple-choice questions, as well as open-ended questions, are commonly used initially [4]. However, these types of questions have limitations when it comes to assessing a student’s analysis and clinical reasoning [5]. To address this, script concordance tests (SCTs) can be used to enhance the development of higher-level clinical reasoning skills [6].

### The Use of SCTs

Methods such as SCTs are grounded in clinical cases designed to mirror real-life clinical scenarios, where information may be incomplete or unclear. The process involves presenting an initial vignette with some preliminary hypotheses, followed by additional information given to the student. SCTs assess how this new information influences the likelihood of the initial hypotheses being considered as correct or relevant [6]. Students express the impact on the initial hypothesis using a 5-level Likert scale ranging from “much less likely” to “much more likely.” This process serves as a proxy for clinical reasoning, aiming to replicate decision-making in actual clinical practice. Typically, specialists in the subject develop the cases, and a robust SCT should comprise a minimum of 60 questions for strong internal validity [7-9]. The student’s responses are then compared to those of an expert panel, ideally consisting of at least 10 experts. Research suggests that 15 experts are necessary for high-impact testing, with minimal added benefit beyond 20 experts [10]. A notable limitation of SCTs is acceptability; a study on SCT acceptability with surgical residents revealed that experts tend to be more satisfied than students. Experts found the questions to be representative of real-life clinical settings [11]. However, SCTs may potentially provide a more precise assessment of students’ clinical reasoning compared to multiple-choice questions [12]. In psychiatry, the use of SCTs is emerging. Early data indicate good internal validity, with a correlation between learners’ education level, test scores, and improvement in evaluations tested before and after a psychiatry rotation [13].

The creation of SCTs demands a substantial investment of human resources [14]. Moreover, the questions are influenced by the designers’ inherent biases, necessitating multiple rounds of refinement with field experts [15]. This iterative process can lead to delays in developing educational materials. In a time when efficiency is crucial—such as during the COVID-19 pandemic or in situations with limited teaching resources—swift adaptations and improvements in the effectiveness of certain teaching methods may be imperative to uphold the quality of medical training [16,17].

### Large Language Models and Their Uses in SCT Design

For clinician-educators seeking assistance in crafting educational materials, recent advancements include the availability of generative artificial intelligence (AI) tools, including large language models (LLM) such as ChatGPT (OpenAI) [18,19]. Originally designed for the public, these tools are currently under scrutiny by various companies and educational institutions to assess their limitations and advantages [20]. Numerous studies highlight the tool’s utility in developing clinical vignettes within medical studies and other health science domains [21]. However, to date, there is no study demonstrating the educational quality of SCT vignettes produced using ChatGPT. Before integrating tools such as ChatGPT into the design of educational materials, it is crucial to evaluate the quality of scenarios, questions, and related expertise generated by ChatGPT, as well as its ability to assess clinical reasoning. It is equally important to consider the potential limitations in using such tools for medical education material design. Although these generative models can be beneficial, they may also introduce errors that limit their usefulness [18]. As for medical students’ attitude toward AI, a recent study on the subject reported that medical students viewed AI in medicine as reliable, trustworthy, and technically competent, although they expressed limited confidence in its capabilities. While acknowledging AI’s intelligence, they did not consider it to be anthropomorphic. The consensus was that fundamental AI knowledge, covering its operation, ethics, applications, reliability, and potential risks, should be integrated into medical education [22].

### Objective and Hypotheses

The primary goal of this project is to investigate how SCTs generated by ChatGPT compare to those produced by clinical experts in 3 key aspects: the scenario (stem), clinical questions, and expert opinion. A secondary objective is to assess whether blind evaluators can distinguish between an SCT generated by ChatGPT and one crafted by experts. Additionally, another subobjective aims to identify the advantages and limitations of the clinical vignettes under examination. Our hypothesis posits that the clinical SCTs created by ChatGPT will likely be considered acceptable by the medical community in terms of scenarios and clinical questions. However, we anticipate that their use with learners may necessitate supervision from clinical experts. Preliminary studies have indicated that AI is a promising tool to aid clinician-educators in designing clinical scenarios. Still, given that the underlying algorithms rely on potentially erroneous data, it is crucial to validate and fine-tune the content before using them as educational materials for learners.

## Methods

### Ethical Considerations

This study received the approval of the ethics of research committee of the Université de Montréal (approval 2023-4906). Participants were given a description of the study in the letter they received and were asked for their consent for their data to be used. Data were anonymized. The participants received no compensation for this study.

### Recruitment

The project was aimed at residents and clinician-educators in the field of psychiatry since SCTs are already used in UGME programs. To be included in the study, participants needed to be either clinician-educators in the field of psychiatry or medical residents in psychiatry affiliated with 1 of Québec’s 4 universities that offer UGME programs (McGill University, Université de Montréal, Université de Sherbrooke, and Université Laval). Psychiatrists not involved in an UGME program were excluded. A total of 100 participants were anticipated for this study, according to similar studies to determine whether there were significant differences between clinical vignettes developed by ChatGPT or those developed by experts [23,24]. Convenience sampling was conducted with the help of the departments of psychiatry of the 4 universities listed above, and a letter was sent out by email that includes a link to a survey that contained all the questions from this study.

### Data Collection

A web-based survey, hosted on LimeSurvey (LimeSurvey GmbH), featured 3 SCTs generated by ChatGPT and 3 SCTs previously crafted by experts in the field, currently used in the digital learning environment at the Université de Montréal. The experts consisted of experienced psychiatrists and primary care physicians who underwent training in SCT concepts. As the primary language for the participants is French, the survey was conducted in French. The original, comprehensive survey in French is available in Multimedia Appendix 1, with an English translation provided in Multimedia Appendix 2. Participants assessed the SCTs based on their respective roles. Due to the anonymous nature of the survey and the inclusion criteria requiring respondents to be either psychiatry residents or physicians, additional demographic data were not collected. The study did, however, document information on the participants’ level of training (resident doctors vs clinician-educators) and their level of clinical experience (0-5, 6-10, or ≥10 y).

Each SCT was evaluated by the participants using the conceptual framework developed by Fournier et al [9] for creating SCTs. This conceptual framework provides a general guideline for SCTs. The SCTs involve real-life medical situations, each describing as a short scenario with some uncertainty. To solve the problem presented in each scenario, there are multiple relevant options available for the medical student. Each scenario, along with its questions, is considered an item. The questions are divided into 3 parts. The first part provides a relevant diagnostic or management option. The second part introduces a new clinical finding, such as a physical sign or test result. The third part uses a 5-point Likert scale for examinees to express their decision on how the new finding affects the option, considering direction (positive, negative, or neutral) and intensity. Examinees are tasked with determining the impact of the new information, and the Likert scale is used to capture their decisions, as script theory suggests that clinical reasoning involves qualitative judgments.

Three components are evaluated by this framework when constructing SCTs: the scenario, clinical questions, and expert opinion. The scenario refers to the stem presented by the SCTs. The clinical questions are the individual questions adding a key element to the stem to stimulate clinical reasoning. The expert opinion refers to the opinion of an expert in the field giving a subjective appreciation as to the ability of the SCT to generate clinical reasoning. The elements of this framework are presented in Table 1. A common SCT template was used for both SCTs generated by ChatGPT and the experts in the field to ensure that the presentation of the SCTs does not create bias.

**Table 1.** The script concordance test (SCT) components with their relevant questions as per the framework by Fournier et al [9] for the evaluation and conception of SCTs.

SCT components and questions		Potential answers
**Scenarios**		
	S1. Describes a challenging circumstance, even for experts	Yes or no
	S2. Describes an appropriate situation for test takers	Yes or no
	S3. The scenario is necessary to understand the question and to set the context	Yes or no
	S4. The clinical presentation is typical	Yes or no
	S5. The scenario is well written	Yes or no
**Clinical questions**		
	Q1. The questions are developed using a key element approach	Yes or no
	Q2. In the opinion of experts, the options are relevant	Yes or no
	Q3. The same option is not found in 2 consecutive questions	Yes or no
	Q4. The new information (second column) makes it possible to test the link between the new information and the option (first column) in the context described	Yes or no
	Q5. Likert-scale anchors are clearly defined and unambiguous	Yes or no
	Q6. Questions are expanded to distribute responses equally across all Likert-scale values	Yes or no
	Q7. Questions are designed to provide a balance between low and high variability	Yes or no

### Expert Opinion

The participants needed to state if the SCT was generated (or not) by ChatGPT (single-blinded mode), give their main hypothesis as to the main diagnosis studied in the SCT, and state in free-text style the strengths and weaknesses of each SCT.

### Creating SCTs With ChatGPT

The ChatGPT tool operates through commands or prompts to enhance its performance. These prompts must offer a context of use, an expertise level, and a specific task. Following the typical steps involved in creating SCTs, we designed the prompts based on the approach outlined in Fournier et al [9]. In this initial study on the subject, we did not explore different sets of prompts, and the generated SCTs were used without modification.

The following commands were entered into ChatGPT to create the SCTs:

1. Act as an expert in university pedagogy of health sciences, in the field of psychiatry.

2. Also acts as an expert in designing thumbnails by script matching.

3. Generates a script matching vignette that includes three questions for the following diagnosis: (diagnosis name), according to DSM-5.

4. Create questions linked to the vignette which start with if you think of ’’a diagnostic hypothesis” and you find ’’a sign or a symptom’’, this hypothesis is probable or not (from −2 to 2, using a Likert scale)

Choosing the ChatGPT 3.5 algorithm as the main LLM for this task made sense for a few key reasons. This algorithm has a vast knowledge base covering a wide array of medical topics, making it an adequate tool for instructors crafting medical questions for medical students [25]. Its natural language comprehension, used in various medical fields, aids in question development [26]. The model’s flexibility allows educators to create different types of questions to suit various learning styles and assessment methods. Notably, ChatGPT 3.5 supports multiple languages, including French, making it accessible for instructors in French-speaking regions. The model’s ability to grasp context enables the creation of questions that build on existing knowledge, providing a more cohesive learning experience [27]. Educators can save time with the model’s human-like text generation based on specific prompts or instructions. It is also crucial to highlight that this algorithm is open access and free, a substantial consideration when cost is a factor in choosing educational tools. Additionally, it is noteworthy that generating an SCT takes less than a minute on average with this tool.

### Selecting Existing Expert-Created SCTs

Three SCTs were chosen at random from the 10 SCTs currently available to learners on the digital learning platform for the clinical psychiatry clerkship rotation at Université de Montréal. As stated above, a total of 3 ChatGPT-generated SCTs and 3 expert-created SCTs were chosen to limit the possibility that chance alone would identify the SCTs generated by ChatGPT from those produced by experts.

### Statistical Analysis

A combined mixed method analysis was conducted with qualitative and quantitative components.

#### Qualitative Analysis

We conducted a content analysis by examining participants’ open responses regarding the advantages and drawbacks of the presented SCTs. The objective was to pinpoint the primary types of benefits and limitations for emphasis. After receiving the open-ended survey responses, we individually extracted emergent themes from respondents using the grounded theory design framework [28]. Subsequently, AH and MP created an initial classification scheme based on these emerging themes. They applied this scheme to annotate the open-ended responses using the Qualitative Data Analysis Miner program (Provalis Research). Any discrepancies in annotations among responders were deliberated upon until a consensus was reached.

#### Quantitative Analysis

We conducted a descriptive statistical analysis to showcase the proportion of participants accurately identifying SCTs generated by ChatGPT compared to those crafted by experts. This same approach was applied to diagnostic hypotheses.

Additionally, we performed a descriptive statistical analysis to compare SCT scores based on the domains of the scenario and clinical questions, following the conceptual framework by Fournier et al [9]. Using a *χ*^2^ test, we assessed the average results within each domain for the SCTs generated by ChatGPT and those by the experts. This allowed us to observe any statistical differences in the responses (yes or no) for various criteria within the scenario and clinical questions domains. We established a statistical significance threshold of *P*<.05 to identify noteworthy observations between the 2 types of SCTs.

## Results

### Participants Characteristics

A total of 102 participants completed the survey. Considering that there are an estimated 400 teaching clinicians in psychiatry in Quebec (about a third of the 1200 practicing psychiatrists), as well as 235 medical residents in psychiatry, this represents 16.1% (102/635) of the pool of potential responders. From the 102 participants, 45 (44.1%) identified as medical residents in psychiatry, 2 (2%) identified as teaching psychiatrists with less than 5 years of experience, 16 (15.7%) identified as teaching psychiatrists with between 6 and 10 years of experience, and 39 (38.2%) identified as teaching psychiatrists with more than 10 years of experience.

### SCT Evaluation

The pooled averages of evaluations of the SCTs for each domain of assessment, stratified by the respondent categories, are shown in Table 2. A complete table reporting the evaluations of the respondents for each individual component of the domains of assessment is available in Multimedia Appendix 3. SCTs 2, 3 and 4 were generated by ChatGPT. It can be observed that there was no significant distinction between the pooled results for the SCTs generated by ChatGPT as compared to those generated by experts in the field. The questions related to the scenario component of the SCTs received better approval from the participants as compared to the clinical questions component.

**Table 2.** Responses for every component of the script concordance test (SCT) evaluations for the 6 SCTs, stratified by respondent categories. “Yes” indicates that the respondents agreed that the domain was elaborated appropriately.

SCT and evaluated component		Medical residents (n=45), n (%)	Teaching physicians (≤5 y; n=2), n (%)	Teaching physicians (6-10 y; n=16), n (%)	Teaching physicians (≥10 y; n=39), n (%)	Pooled average(N=102), n (%)
**SCT 1**						
	Scenario (yes)	30 (67)	2 (100)	12 (75)	31 (79)	75 (74)
	Clinical questions (yes)	29 (64)	2 (100)	13 (81)	28 (72)	72 (71)
	Is it a ChatGPT-generated scenario? (correct answers)	25 (44)	1 (50)	6 (38)	18 (54)	50 (49)
**SCT 2** ^**a**^						
	Scenario (yes)	29 (64)	2 (100)	13 (81)	25 (64)	69 (68)
	Clinical questions (yes)	30 (67)	2 (100)	14 (88)	25 (64)	71 (70)
	Is it a ChatGPT-generated scenario? (correct answers)	22 (49)	0 (0)	6 (38)	18 (46)	46 (45)
**SCT 3** ^**a**^						
	Scenario (yes)	28 (62)	2 (100)	12 (75)	26 (67)	68 (67)
	Clinical questions (yes)	28 (62)	2 (100)	13 (81)	25 (64)	68 (67)
	Is it a ChatGPT-generated scenario? (correct answers)	16 (36)	0 (0)	4 (25)	16 (41)	36 (35)
**SCT 4** ^**a**^						
	Scenario (yes)	28 (62)	2 (100)	11 (69)	26 (67)	67 (66)
	Clinical questions (yes)	25 (56)	2 (100)	14 (88)	28 (72)	69 (68)
	Is it a ChatGPT-generated scenario? (correct answers)	19 (42)	1 (50)	6 (38)	12 (31)	38 (37)
**SCT 5**						
	Scenario (yes)	26 (58)	2 (100)	11 (69)	26 (67)	65 (64)
	Clinical questions (yes)	27 (60)	2 (100)	13 (81)	28 (72)	70 (69)
	Is it a ChatGPT-generated scenario? (correct answers)	21 (53)	2 (100)	8 (50)	23 (59)	54 (53)
**SCT 6**						
	Scenario (yes)	27 (60)	2 (100)	12 (75)	26 (67)	67 (66)
	Clinical questions (yes)	24 (53)	2 (100)	13 (81)	27 (69)	66 (65)
	Is it a ChatGPT-generated scenario? (correct answers)	21 (53)	1 (50)	8 (50)	18 (46)	48 (47)

a Script concordance tests created by ChatGPT.

Participants could not identify which SCT was created by ChatGPT from those created by experts in the field, as observed in Table 2. Teaching clinicians with more than 10 years of experience tended to better recognize SCTs generated by ChatGPT than their peers with less experience and medical residents, except for SCT 4.

### Comparisons Between ChatGPT- and Expert-Generated SCTs

When using the pooled observations for the scenario and clinical questions domains across the SCTs generated by ChatGPT and those generated by experts, no statistically significant distinctions were observed when comparing both types of SCTs (all *P*>.05), as seen in Table 3.

**Table 3.** Comparisons of the script concordance tests (SCTs) generated by ChatGPT as opposed to those generated by experts in the field.

Components	SCTs 1, 5, and 6 (experts), average score (%)	SCTs 2, 3, and 4 (ChatGPT), average score (%)	*P* value (ChatGPT-generated vs expert-generated SCTs)
Scenario	66.40	67.27	.84
Clinical questions	70.05	68.86	.99
Identifying if generated by AI^a^	54	40	.07

a AI: artificial intelligence.

### Reported Strengths and Weaknesses of the SCTs

#### Overview

Only 39 (38.2%) of the 102 participants wrote at least 1 comment on the strengths or weaknesses for each of individual SCT. The strengths and weaknesses of the SCTs generated by ChatGPT were similarly reported across all the respondents and resembled those identified for the SCTs generated by experts in the field. Respondents reported that SCTs generated by ChatGPT were well aligned with the *Diagnostic and Statistical Manual of Mental Disorders, Fifth Edition* (*DSM-5*) but were also too caricatural.

#### Strengths of the SCTs Generated by Experts in the Field

Overall, 3 (8%) of the 39 respondents indicated for 1 or more SCTs generated by experts in the field that the scenario represented typical clinical challenges. Most of the respondents (27/39, 69%) reported that the SCTs used clear prompts to test clinical reasoning. Sample responses included the following:

*This concordance test was easy to follow as because the scenarios were concise and the prompts were clear.* [Respondent 1]

*In terms of clarity, the prompts were well written and it was very simple to see how they could elicit clinical reasoning.* [Respondent 9]

#### Strengths of the SCTs Generated by ChatGPT

Almost all respondents (32/39, 82%) mentioned that the SCTs were using typical clinical signs and symptoms reported in the *DSM-5*. Some (5/39, 13%) indicated that the SCTs were very well nuanced. Sample responses included the following:

*This scenario corresponds to the textbook’s description of the presented diagnosis.* [Respondent 4]

*I see that these prompts do not try to derive too much from the differential diagnoses intended for the suggested clinical presentation. They offered a degree of flexibility to enable the student to use their clinical reasoning.* [Respondent 71]

#### Limitations of the SCTs Generated by Experts in the Field

In all, 2 (5%) of the 39 respondents mentioned that they found the SCTs straightforward and unchallenging. There were no other comments regarding the limitations of the SCTs generated by experts in the field. Sample responses included the following:

*This scenario is too easy. I find little value as it is clear for the student that we are looking at the specific diagnosis.* [Respondent 1]

*I don’t see how this is challenging for the medical student who is going to take this test.* [Respondent 80]

#### Limitations of the SCTs Generated by ChatGPT

Most respondents (29/39, 74%) reported the SCTs generated by ChatGPT as caricatural or stereotypical clinical presentations as observed in textbooks with little regard to atypical presentations. A total of 7 (18%) respondents indicated that the SCTs generated by ChatGPT were too simple, as they tended to include additional information that were too trivial when attempting to challenge the responder’s clinical reasoning. Sample responses included the following:

*This is very trivial. I mean, it is not very difficult to find out what are the answers to these prompts as they clearly hint towards the same diagnosis.* [Respondent 3]

*It would be interesting to add more challenging prompts as they tend to be very simplistic and poorly represent complex clinical cases as they are very stereotypical to what is found in the DSM-5.* [Respondent 4]

## Discussion

### Principal Findings

The aim of this study was to compare SCTs created by ChatGPT to SCTs produced by clinical specialists on the scenario (stem), clinical questions, and expert opinions. There were no significant distinctions between the SCTs generated by ChatGPT as compared to those developed by experts in the field for the evaluated components. The strengths and weaknesses were similar across the 2 types of SCT. Respondents reported that the SCTs generated by ChatGPT were well aligned with the *DSM-5* but were also too caricatural.

### Comparison With Prior Work

Since the creation of ChatGPT, it has been used in various areas of medical education such as digital teaching assistants and personalized education [29]. As a recent exploration study on the role of LLMs such as ChatGPT demonstrated, these models can provide interactive cases in a medical education context [30]. Considering these previous studies of ChatGPT in the development of medical education tools, it is possible that the inability to recognize a SCT generated by ChatGPT from one developed by experts in the field can be explained by the generative nature of this LLM. As such, a recent review on the use of ChatGPT in health care has identified that this form of AI can be used for problem-based learning and critical thinking in health care education [31]. However, it is mentioned in the literature that although the quality of the scenarios (or information) generated by ChatGPT might appear impressive, there is a need for an expert to assess the content generated, as it might be an amalgamation of erroneous information [32].

Although a few comments were provided regarding the strengths and limitations of both types of SCTs, they align with what is commonly reported in the literature for similar tasks. Some respondents noted caricature-like scenarios, possibly attributed to the robotic and dehumanized nature often associated with vignettes produced by LLMs [33]. It is plausible that more intricate prompts could have resulted in more nuanced scenarios. Therefore, the mentioned strengths of the scenarios and clinical questions, particularly their clinical alignment with the *DSM-5*, may be tied to the fact that this was one of the prompts used when conceptualizing interactions with ChatGPT during the creation of the SCTs.

In the field of psychiatry, applications of ChatGPT to medical education are limited. Among the limited available evidence, a novel study tested the knowledge of ChatGPT by exposing it to 100 clinical cases vignettes, and it performed extremely well [34]. Another similar use of ChatGPT was as an aid to answer clinical questions. A recent study evaluated the performance of users (psychiatrist and medical residents in the Netherlands) using ChatGPT as compared to nonusers for answering several questions in psychiatry, and it was observed that the users had better and faster responses as compared to nonusers [35]. Although these applications differ from this study, they might hint that ChatGPT currently has a database that holds relevant data in the field of psychiatry, which might explain the realism of scenarios and prompts observed for SCTs 2, 3, and 4.

There are substantial ethical considerations that must be accounted for when using such tool to assist medical educators. As an example, it is important to consider that ChatGPT (and other LLMs) are bound to the data they have been trained with along with their inherent biases [36]. Cross-validation of the generated information is often necessary to ensure that learners are not exposed to false information [37].

### Limitations

Although web-based surveys offer convenience in distribution, they struggle with the challenge of accurately identifying the characteristics of the assessed population [38]. In our survey, we did not differentiate between those formally trained in SCTs and those who merely encountered them during their medical training, thus introducing potential limitations in generalizing the results. It is plausible that clinicians more experienced with SCTs were more likely to participate in the survey, but our recruitment from psychiatry departments exclusively helps mitigate this bias. Interpretation biases may also be present, as not all participants might be familiar with the framework used in this study. We did not explore acceptability regarding the use of generative AI in SCT creation, marking another limitation. Additionally, we did not compare different prompts, and it is conceivable that alternative sets of prompts could have produced better results for the SCTs generated by ChatGPT. Opting for a different language model might have yielded varied performances, and it is plausible that alternative models could outperform ChatGPT in this context.

### Conclusions

In an era of rapidly evolving medicine and where technologies derived from AI are growing even more quickly, this study is the first to focus on the design of SCTs assisted by AI. The primary goal of this study highlighted that no statistical differences were found between the SCTs generated by ChatGPT and those created by clinical experts in the field of psychiatry for the elaboration of a scenario and the clinical questions presented in the SCTs. On average, the respondents incorrectly identified which SCTs were created with the help of AI. The major strength of SCTs generated by ChatGPT was that they were consistent with the *DSM-5*, whereas the caricatural quality or triviality of the SCTs generated by ChatGPT were the main weaknesses reported by the respondents. A possible way to mitigate this effect would be to provide more complex prompts to the generative AI or editing some details of the vignette. This study opens the door to larger-scale studies in this area to assess the impact of such aid on the academic success of medical students and how it can be used to improve efficiencies.

## Supplementary material

10.2196/54067 Multimedia Appendix 1 Original survey in French.

10.2196/54067 Multimedia Appendix 2 Translated survey in English.

10.2196/54067 Multimedia Appendix 3 Responses for every component of the script concordance test (SCT) evaluations for the 6 SCTs, stratified by the category of respondents.

## Abbreviations

AI: artificial intelligence
DSM-5: Diagnostic and Statistical Manual of Mental Disorders, Fifth Edition
LLM: large language model
SCT: script concordance test
UGME: undergraduate medical education
