# Supplementary material for: Using ChatGPT in Psychiatry to Design Script Concordance Tests in Undergraduate Medical Education: Mixed Methods Study
Source: JMIR Med Educ. 2024 Apr 4;10:e54067. doi: 10.2196/54067 (PMC11007379; doi:10.2196/54067)
Supplement: Multimedia Appendix 1 [file mededu-v10-e54067-s001.pdf]

ChatGPT comme outil d'aide à la conception de matériel pédagogique en psychiatrie

Merci de prendre le temps de répondre au questionnaire suivant.

Celui-ci comporte 6 vignettes cliniques dont certaines ont été générées par intelligence artificielle (ChatGPT). Nous vous demandons d'évaluer les vignettes cliniques (pour l'enseignement-apprentissage de la psychiatrie) selon des critères bien précis.

Le temps de complétion du questionnaire est estimé entre 5 et 15 minutes.

Il y a 20 questions dans ce questionnaire.

Informations sociodémographiques

Quel est votre niveau de formation?

Veuillez sélectionner une réponse ci-dessous
Veuillez sélectionner une seule des propositions suivantes :

- ◯ Médecin résident.e en psychiatrie
- ◯ Clinicien-enseignant / Psychiatre

Quel est votre nombre d'années d'expériences en clinique (incluant la résidence)?

Veuillez sélectionner une réponse ci-dessous
Veuillez sélectionner une seule des propositions suivantes :

- ◯ 0 à 5 ans
- ◯ 6 à 10 ans
- ◯ Plus de 10 ans

Vignette 1

Voici la première vignette. Merci de la lire attentivement .

Situation professionnelle authentique :

Julien, 22 ans, se présente à l'urgence psychiatrique. Il souligne avoir beaucoup de difficulté à se concentrer dans ses études et la réalisation de ses travaux scolaires depuis quelques semaines. Facilement distrait, il lui arrive d'être fréquemment dans la lune.

Si vous pensiez ...	Et qu'alors...	Votre hypothèse en est ...
**1- À une dépression majeure**	Monsieur soulève avoir une fatigue importante durant la journée et se réveille presqu'à toutes les nuits vers 3AM.	☐ Fortement affaiblie<br>☐ Affaiblie<br>☐ Inchangée<br>☐ Renforcée<br>☐ Fortement renforcée
**2- À un trouble d'anxiété généralisée**	Monsieur dit être convaincu d'avoir une maladie grave.	☐ Fortement affaiblie<br>☐ Affaiblie<br>☐ Inchangée<br>☐ Renforcée<br>☐ Fortement renforcée
**3- À un trouble déficitaire de l'attention**	Monsieur était premier de classe au primaire.	☐ Fortement affaiblie<br>☐ Affaiblie<br>☐ Inchangée<br>☐ Renforcée<br>☐ Fortement renforcée

Situation professionnelle authentique :

Julien, 22 ans, se présente à l'urgence psychiatrique. Il souligne avoir beaucoup de difficulté à se concentrer dans ses études et la réalisation de ses travaux scolaires depuis quelques semaines. Facilement distrait, il lui arrive d'être fréquemment dans la lune.

Si vous pensiez ...	Et qu'alors...	Votre hypothèse en est ...
**1- À une dépression majeure**	Monsieur soulève avoir une fatigue importante durant la journée et se réveille presqu'à toutes les nuits vers 3AM.	☐ Fortement affaiblie<br>☐ Affaiblie<br>☐ Inchangée<br>☐ Renforcée<br>☐ Fortement renforcée
**2- À un trouble d'anxiété généralisée**	Monsieur dit être convaincu d'avoir une maladie grave.	☐ Fortement affaiblie<br>☐ Affaiblie<br>☐ Inchangée<br>☐ Renforcée<br>☐ Fortement renforcée
**3- À un trouble déficitaire de l'attention**	Monsieur était premier de classe au primaire.	☐ Fortement affaiblie<br>☐ Affaiblie<br>☐ Inchangée<br>☐ Renforcée<br>☐ Fortement renforcée

Choisissez la réponse appropriée pour chaque élément :

	Oui	Incertain	Non
**La vignette décrit une situation difficile, même pour les experts**	◯	◯	◯
**La vignette décrit une situation appropriée pour des étudiants en médecine**	◯	◯	◯
**Le scénario est nécessaire pour comprendre la question et pour situer le contexte**	◯	◯	◯
**La présentation clinique est typique**	◯	◯	◯
**Le scénario est correctement écrit**	◯	◯	◯
**Les questions sont élaborées selon une approche par éléments clés (ajout d'un élément clinique important)**	◯	◯	◯
**À votre avis, les options (questions proposées) sont pertinentes**	◯	◯	◯
**La même option (ex.: diagnostic) ne se retrouve pas dans deux questions consécutives**	◯	◯	◯
**La nouvelle information (2ème colonne) permet de tester le lien entre la nouvelle information et l'option (1ère colonne) dans le contexte décrit**	◯	◯	◯

	Oui	Incertain	Non
**Les ancres d'échelle de Likert (dernière colonne) sont clairement définies et sans ambiguïté**	◯	◯	◯
**Les questions sont développées pour répartir les réponses de manière égale sur toutes les valeurs de l'échelle de Likert**	◯	◯	◯
**Les questions sont élaborées pour fournir un équilibre entre la variabilité faible et élevée (entre les questions)**	◯	◯	◯
**Selon vous, la vignette a-t-elle été réalisée à partir de ChatGPT?**	◯	◯	◯

Quel est selon-vous le diagnostic principal réflété par cette vignette clinique? *

Veuillez écrire votre réponse ici :

Quelles sont les forces et les faiblesses de la vignette? *

Veuillez écrire votre réponse ici :

Vignette 2

Voici la deuxième vignette. Merci de la lire attentivement .

Situation professionnelle authentique :

M. Dupont, âgé de 35 ans, est un vétéran de l'armée. Il présente des cauchemars récurrents, des flashbacks et une forte anxiété lorsqu'il entend des bruits similaires à ceux de tirs d'armes à feu.

Si vous pensiez ...	Et qu'alors...	Votre hypothèse en est ...
**1- À un trouble stress post-traumatique**	Monsieur mentionne éviter les situations qui lui rappelle les armes à feu	☐ Fortement affaiblie<br>☐ Affaiblie<br>☐ Inchangée<br>☐ Renforcée<br>☐ Fortement renforcée
**2- À un trouble dépressif caractérisé**	Monsieur dit être avoir de graves problèmes de concentration	☐ Fortement affaiblie<br>☐ Affaiblie<br>☐ Inchangée<br>☐ Renforcée<br>☐ Fortement renforcée
**3- À un trouble d'adaptation avec humeur anxieuse**	Monsieur mentionne que l'anxiété est aussi intense dans les autres sphères de sa vie	☐ Fortement affaiblie<br>☐ Affaiblie<br>☐ Inchangée<br>☐ Renforcée<br>☐ Fortement renforcée

Situation professionnelle authentique :

M. Dupont, âgé de 35 ans, est un vétéran de l'armée. Il présente des cauchemars récurrents, des flashbacks et une forte anxiété lorsqu'il entend des bruits similaires à ceux de tirs d'armes à feu.

Si vous pensiez ...	Et qu'alors...	Votre hypothèse en est ...
**1- À un trouble stress post-traumatique**	Monsieur mentionne éviter les situations qui lui rappelle les armes à feu	☐ Fortement affaiblie<br>☐ Affaiblie<br>☐ Inchangée<br>☐ Renforcée<br>☐ Fortement renforcée
**2- À un trouble dépressif caractérisé**	Monsieur dit être avoir de graves problèmes de concentration	☐ Fortement affaiblie<br>☐ Affaiblie<br>☐ Inchangée<br>☐ Renforcée<br>☐ Fortement renforcée
**3- À un trouble d'adaptation avec humeur anxieuse**	Monsieur mentionne que l'anxiété est aussi intense dans les autres sphères de sa vie	☐ Fortement affaiblie<br>☐ Affaiblie<br>☐ Inchangée<br>☐ Renforcée<br>☐ Fortement renforcée

*

Choisissez la réponse appropriée pour chaque élément :

	Oui	Incertain	Non
**La vignette décrit une situation difficile, même pour les experts**	○	○	○
**La vignette décrit une situation appropriée pour des étudiants en médecine**	○	○	○
**Le scénario est nécessaire pour comprendre la question et pour situer le contexte**	○	○	○
**La présentation clinique est typique**	○	○	○
**Le scénario est correctement écrit**	○	○	○
**Les questions sont élaborées selon une approche par éléments clés (ajout d'un élément clinique important)**	○	○	○
**À votre avis, les options (questions proposées) sont pertinentes**	○	○	○
**La même option (ex.: diagnostic) ne se retrouve pas dans deux questions consécutives**	○	○	○
**La nouvelle information (2ème colonne) permet de tester le lien entre la nouvelle information et**	○	○	○

	Oui	Incertain	Non
**l'option (1ère colonne) dans le contexte décrit**			
**Les ancres d'échelle de Likert (dernière colonne) sont clairement définies et sans ambiguïté**	○	○	○
**Les questions sont développées pour répartir les réponses de manière égale sur toutes les valeurs de l'échelle de Likert**	○	○	○
**Les questions sont élaborées pour fournir un équilibre entre la variabilité faible et élevée (entre les questions)**	○	○	○
**Selon vous, la vignette a-t-elle été réalisée à partir de ChatGPT?**	○	○	○

Quel est selon-vous le diagnostic principal reflété par cette vignette clinique? *

Veuillez écrire votre réponse ici :

Quelles sont les forces et les faiblesses de la vignette? *

Veuillez écrire votre réponse ici :

Vignette 3

Voici la troisième vignette. Merci de la lire attentivement .

Situation professionnelle authentique :

Sophie est une femme âgée de 28 ans, est comptable. Elle passe la plupart de son temps libre à vérifier constamment les portes et les fenêtres de sa maison, craignant qu'elles ne soient pas correctement verrouillées. Elle se lave également les mains à répétition, souvent jusqu'à ce qu'elles soient rouges et irritées.

Si vous pensiez ...	Et qu'alors...	Votre hypothèse en est ...
**1- À un trouble d'anxiété généralisé**	Sophie mentionne que ses préoccupations sont seulement lorsqu'elle est chez elle	☐ Fortement affaiblie<br>☐ Affaiblie<br>☐ Inchangée<br>☐ Renforcée<br>☐ Fortement renforcée
**2- À un trouble de la personnalité obsessionnelle-compulsive**	Sophie rapporte être perfectionniste de nature	☐ Fortement affaiblie<br>☐ Affaiblie<br>☐ Inchangée<br>☐ Renforcée<br>☐ Fortement renforcée
**3- À un trouble obsessionnel-compulsif**	Sophie dit que ses symptômes interfèrent avec son fonctionnement quotidien	☐ Fortement affaiblie<br>☐ Affaiblie<br>☐ Inchangée<br>☐ Renforcée<br>☐ Fortement renforcée

Situation professionnelle authentique :

Sophie est une femme âgée de 28 ans, est comptable. Elle passe la plupart de son temps libre à vérifier constamment les portes et les fenêtres de sa maison, craignant qu'elles ne soient pas correctement verrouillées. Elle se lave également les mains à répétition, souvent jusqu'à ce qu'elles soient rouges et irritées.

Si vous pensiez …	Et qu'alors…	Votre hypothèse en est …
**1- À un trouble d'anxiété généralisé**	Sophie mentionne que ses préoccupations sont seulement lorsqu'elle est chez elle	☐ Fortement affaiblie<br>☐ Affaiblie<br>☐ Inchangée<br>☐ Renforcée<br>☐ Fortement renforcée
**2- À un trouble de la personnalité obsessionnelle-compulsive**	Sophie rapporte être perfectionniste de nature	☐ Fortement affaiblie<br>☐ Affaiblie<br>☐ Inchangée<br>☐ Renforcée<br>☐ Fortement renforcée
**3- À un trouble obsessionnel-compulsif**	Sophie dit que ses symptômes interfèrent avec son fonctionnement quotidien	☐ Fortement affaiblie<br>☐ Affaiblie<br>☐ Inchangée<br>☐ Renforcée<br>☐ Fortement renforcée

*

Choisissez la réponse appropriée pour chaque élément :

	Oui	Incertain	Non
**La vignette décrit une situation difficile, même pour les experts**	○	○	○
**La vignette décrit une situation appropriée pour des étudiants en médecine**	○	○	○
**Le scénario est nécessaire pour comprendre la question et pour situer le contexte**	○	○	○
**La présentation clinique est typique**	○	○	○
**Le scénario est correctement écrit**	○	○	○
**Les questions sont élaborées selon une approche par éléments clés (ajout d'un élément clinique important)**	○	○	○
**À votre avis, les options (questions proposées) sont pertinentes**	○	○	○
**La même option (ex.: diagnostic) ne se retrouve pas dans deux questions consécutives**	○	○	○
**La nouvelle information (2ème colonne) permet de tester le lien entre la nouvelle information et**	○	○	○

	Oui	Incertain	Non
**l'option (1ère colonne) dans le contexte décrit**			
**Les ancres d'échelle de Likert (dernière colonne) sont clairement définies et sans ambiguïté**	◯	◯	◯
**Les questions sont développées pour répartir les réponses de manière égale sur toutes les valeurs de l'échelle de Likert**	◯	◯	◯
**Les questions sont élaborées pour fournir un équilibre entre la variabilité faible et élevée (entre les questions)**	◯	◯	◯
**Selon vous, la vignette a-t-elle été réalisée à partir de ChatGPT?**	◯	◯	◯

Quel est selon-vous le diagnostic principal réflété par cette vignette clinique? *

Veuillez écrire votre réponse ici :

Quelles sont les forces et les faiblesses de la vignette? *

Veuillez écrire votre réponse ici :

Vignette 4

Voici la quatrième vignette. Merci de la lire attentivement .

Situation professionnelle authentique :

Marie a 34 ans. Elle consulte fréquemment les médecins pour des symptômes variés tels que des douleurs abdominales, des maux de tête et des éruptions cutanées. Malgré des examens approfondis, aucune cause médicale sous-jacente n'est trouvée. Elle a également été hospitalisée plusieurs fois pour des symptômes graves, mais aucun diagnostic médical concret n'a été établi.

Si vous pensiez ...	Et qu'alors...	Votre hypothèse en est ...
**1- À un trouble factice**	Marie mentionne travailler comme infirmière dans un hôpital communautaire	☐ Fortement affaiblie<br>☐ Affaiblie<br>☐ Inchangée<br>☐ Renforcée<br>☐ Fortement renforcée
**2- À un trouble à symptomatologie somatique**	Marie présente par moment une paralysie du membre supérieur droit	☐ Fortement affaiblie<br>☐ Affaiblie<br>☐ Inchangée<br>☐ Renforcée<br>☐ Fortement renforcée
**3- À de la simulation**	Marie mentionne vouloir obtenir un statut d'invalidité afin de ne plus avoir à travailler	☐ Fortement affaiblie<br>☐ Affaiblie<br>☐ Inchangée<br>☐ Renforcée<br>☐ Fortement renforcée

Situation professionnelle authentique :

Marie a 34 ans. Elle consulte fréquemment les médecins pour des symptômes variés tels que des douleurs abdominales, des maux de tête et des éruptions cutanées. Malgré des examens approfondis, aucune cause médicale sous-jacente n'est trouvée. Elle a également été hospitalisée plusieurs fois pour des symptômes graves, mais aucun diagnostic médical concret n'a été établi.

Si vous pensiez ...	Et qu'alors...	Votre hypothèse en est ...
**1- À un trouble factice**	Marie mentionne travailler comme infirmière dans un hôpital communautaire	☐ Fortement affaiblie<br>☐ Affaiblie<br>☐ Inchangée<br>☐ Renforcée<br>☐ Fortement renforcée
**2- À un trouble à symptomatologie somatique**	Marie présente par moment une paralysie du membre supérieur droit	☐ Fortement affaiblie<br>☐ Affaiblie<br>☐ Inchangée<br>☐ Renforcée<br>☐ Fortement renforcée
**3- À de la simulation**	Marie mentionne vouloir obtenir un statut d'invalidité afin de ne plus avoir à travailler	☐ Fortement affaiblie<br>☐ Affaiblie<br>☐ Inchangée<br>☐ Renforcée<br>☐ Fortement renforcée

*

Choisissez la réponse appropriée pour chaque élément :

	Oui	Incertain	Non
**La vignette décrit une situation difficile, même pour les experts**	◯	◯	◯
**La vignette décrit une situation appropriée pour des étudiants en médecine**	◯	◯	◯
**Le scénario est nécessaire pour comprendre la question et pour situer le contexte**	◯	◯	◯
**La présentation clinique est typique**	◯	◯	◯
**Le scénario est correctement écrit**	◯	◯	◯
**Les questions sont élaborées selon une approche par éléments clés (ajout d'un élément clinique important)**	◯	◯	◯
**À votre avis, les options (questions proposées) sont pertinentes**	◯	◯	◯
**La même option (ex.: diagnostic) ne se retrouve pas dans deux questions consécutives**	◯	◯	◯
**La nouvelle information (2ème colonne) permet de tester le lien entre la nouvelle information et**	◯	◯	◯

	Oui	Incertain	Non
**l'option (1ère colonne) dans le contexte décrit**			
**Les ancres d'échelle de Likert (dernière colonne) sont clairement définies et sans ambiguïté**	◯	◯	◯
**Les questions sont développées pour répartir les réponses de manière égale sur toutes les valeurs de l'échelle de Likert**	◯	◯	◯
**Les questions sont élaborées pour fournir un équilibre entre la variabilité faible et élevée (entre les questions)**	◯	◯	◯
**Selon vous, la vignette a-t-elle été réalisée à partir de ChatGPT?**	◯	◯	◯

Quel est selon-vous le diagnostic principal reflété par cette vignette clinique? *

Veuillez écrire votre réponse ici :

Quelles sont les forces et les faiblesses de la vignette? *

Veuillez écrire votre réponse ici :

Vignette 5

Voici la cinquième vignette. Merci de la lire attentivement .

Situation professionnelle authentique :

Vous êtes demandés à l'étage en consultation pour Monsieur Tremblay 55 ans pour agitation sévère. Vous apprenez qu'il prend du Lithium et du Citalopram (Celexa).

Si vous pensiez ...	Et qu'alors...	Votre hypothèse en est ...
**1- Un syndrome sérotoninergique**	Vous apprenez qu'il a reçu de l'Haldol hier soir.	☐ Fortement affaiblie<br>☐ Affaiblie<br>☐ Inchangée<br>☐ Renforcée<br>☐ Fortement renforcée
**2- Une intoxication au Lithium.**	Vous recevez une alerte au laboratoire pour une créatinine à 300 mmol/L.	☐ Fortement affaiblie<br>☐ Affaiblie<br>☐ Inchangée<br>☐ Renforcée<br>☐ Fortement renforcée
**3- Un délirium**	À l'examen physique vous avez une rigidité musculaire importante aux membres supérieurs et inférieurs.	☐ Fortement affaiblie<br>☐ Affaiblie<br>☐ Inchangée<br>☐ Renforcée<br>☐ Fortement renforcée

Situation professionnelle authentique :

Vous êtes demandés à l'étage en consultation pour Monsieur Tremblay 55 ans pour agitation sévère. Vous apprenez qu'il prend du Lithium et du Citalopram (Celexa).

Si vous pensiez ...	Et qu'alors...	Votre hypothèse en est ...
**1- Un syndrome sérotoninergique**	Vous apprenez qu'il a reçu de l'Haldol hier soir.	☐ Fortement affaiblie<br>☐ Affaiblie<br>☐ Inchangée<br>☐ Renforcée<br>☐ Fortement renforcée
**2- Une intoxication au Lithium.**	Vous recevez une alerte au laboratoire pour une créatinine à 300 mmol/L.	☐ Fortement affaiblie<br>☐ Affaiblie<br>☐ Inchangée<br>☐ Renforcée<br>☐ Fortement renforcée
**3- Un délirium**	À l'examen physique vous avez une rigidité musculaire importante aux membres supérieurs et inférieurs.	☐ Fortement affaiblie<br>☐ Affaiblie<br>☐ Inchangée<br>☐ Renforcée<br>☐ Fortement renforcée

*

Choisissez la réponse appropriée pour chaque élément :

	Oui	Incertain	Non
**La vignette décrit une situation difficile, même pour les experts**	○	○	○
**La vignette décrit une situation appropriée pour des étudiants en médecine**	○	○	○
**Le scénario est nécessaire pour comprendre la question et pour situer le contexte**	○	○	○
**La présentation clinique est typique**	○	○	○
**Le scénario est correctement écrit**	○	○	○
**Les questions sont élaborées selon une approche par éléments clés (ajout d'un élément clinique important)**	○	○	○
**À votre avis, les options (questions proposées) sont pertinentes**	○	○	○
**La même option (ex.: diagnostic) ne se retrouve pas dans deux questions consécutives**	○	○	○
**La nouvelle information (2ème colonne) permet de tester le lien entre la nouvelle information et l'option (1ère colonne) dans le contexte décrit**	○	○	○

	Oui	Incertain	Non
**Les ancres d'échelle de Likert (dernière colonne) sont clairement définies et sans ambiguïté**	◯	◯	◯
**Les questions sont développées pour répartir les réponses de manière égale sur toutes les valeurs de l'échelle de Likert**	◯	◯	◯
**Les questions sont élaborées pour fournir un équilibre entre la variabilité faible et élevée (entre les questions)**	◯	◯	◯
**Selon vous, la vignette a-t-elle été réalisée à partir de ChatGPT?**	◯	◯	◯

Quel est selon-vous le diagnostic principal reflété par cette vignette clinique?

Veuillez écrire votre réponse ici :

Quelles sont les forces et les faiblesses de la vignette? *

Veuillez écrire votre réponse ici :

Vignette 6

Voici la dernière vignette. Merci de la lire attentivement.

Situation professionnelle authentique :

Julie, 26 ans, est amenée à l'urgence par les policiers. Elle est agitée, hurle et mentionne être pourchassée. Mme n'a pas d'antécédents médicaux et ne prend pas de médicament.

Si vous pensiez ...	Et qu'alors...	Votre hypothèse en est ...
**1- À une maladie bipolaire affective de type 1**	Des informations collatérales nous indiquent que Madame ne dort pas depuis 2 jours.	☐ Fortement affaiblie<br>☐ Affaiblie<br>☐ Inchangée<br>☐ Renforcée<br>☐ Fortement renforcée
**2- À un trouble de la personnalité limite**	Madame a tendance à consommer du cannabis, de la cocaine et de l'héroïne de façon impulsive en présence de ses amis.	☐ Fortement affaiblie<br>☐ Affaiblie<br>☐ Inchangée<br>☐ Renforcée<br>☐ Fortement renforcée
**3- À une schizophrénie**	La sœur de Madame est atteinte de schizophrénie.	☐ Fortement affaiblie<br>☐ Affaiblie<br>☐ Inchangée<br>☐ Renforcée<br>☐ Fortement renforcée

Situation professionnelle authentique :

Julie, 26 ans, est amenée à l'urgence par les policiers. Elle est agitée, hurle et mentionne être pourchassée. Mme n'a pas d'antécédents médicaux et ne prend pas de médicament.

Si vous pensiez ...	Et qu'alors...	Votre hypothèse en est ...
**1- À une maladie bipolaire affective de type 1**	Des informations collatérales nous indiquent que Madame ne dort pas depuis 2 jours.	☐ Fortement affaiblie<br>☐ Affaiblie<br>☐ Inchangée<br>☐ Renforcée<br>☐ Fortement renforcée
**2- À un trouble de la personnalité limite**	Madame a tendance à consommer du cannabis, de la cocaine et de l'héroine de façon impulsive en présence de ses amis.	☐ Fortement affaiblie<br>☐ Affaiblie<br>☐ Inchangée<br>☐ Renforcée<br>☐ Fortement renforcée
**3- À une schizophrénie**	La sœur de Madame est atteinte de schizophrénie.	☐ Fortement affaiblie<br>☐ Affaiblie<br>☐ Inchangée<br>☐ Renforcée<br>☐ Fortement renforcée

*

Choisissez la réponse appropriée pour chaque élément :

	Oui	Incertain	Non
**La vignette décrit une situation difficile, même pour les experts**	◯	◯	◯
**La vignette décrit une situation appropriée pour des étudiants en médecine**	◯	◯	◯
**Le scénario est nécessaire pour comprendre la question et pour situer le contexte**	◯	◯	◯
**La présentation clinique est typique**	◯	◯	◯
**Le scénario est correctement écrit**	◯	◯	◯
**Les questions sont élaborées selon une approche par éléments clés (ajout d'un élément clinique important)**	◯	◯	◯
**À votre avis, les options (questions proposées) sont pertinentes**	◯	◯	◯
**La même option (ex.: diagnostic) ne se retrouve pas dans deux questions consécutives**	◯	◯	◯
**La nouvelle information (2ème colonne) permet de tester le lien entre la nouvelle information et l'option (1ère colonne) dans le contexte décrit**	◯	◯	◯

	Oui	Incertain	Non
**Les ancres d'échelle de Likert (dernière colonne) sont clairement définies et sans ambiguïté**	◯	◯	◯
**Les questions sont développées pour répartir les réponses de manière égale sur toutes les valeurs de l'échelle de Likert**	◯	◯	◯
**Les questions sont élaborées pour fournir un équilibre entre la variabilité faible et élevée (entre les questions)**	◯	◯	◯
**Selon vous, la vignette a-t-elle été réalisée à partir de ChatGPT?**	◯	◯	◯

Quel est selon-vous le diagnostic principal reflété par cette vignette clinique?

Veuillez écrire votre réponse ici :

Quelles sont les forces et les faiblesses de la vignette? *

Veuillez écrire votre réponse ici :

Merci beaucoup pour votre temps et d'avoir complété le questionnaire!

Si vous avez des questions sur le projet de recherche ou si vous souhaitez vous retirer du projet de recherche, vous pouvez communiquer avec le chercheur responsable de ce projet de recherche aux coordonnées suivantes : Dr. Alexandre Hudon, médecin résident en psychiatrie et coordonnateur du projet (alexandre.hudon.1@umontreal.ca, 514-995-4842) .

21/10/2023 – 16:41
Envoyer votre questionnaire.
Merci d'avoir complété ce questionnaire.
