# Supplementary material for: Using ChatGPT in Psychiatry to Design Script Concordance Tests in Undergraduate Medical Education: Mixed Methods Study
Source: JMIR Med Educ. 2024 Apr 4;10:e54067. doi: 10.2196/54067 (PMC11007379; doi:10.2196/54067)
Supplement: Multimedia Appendix 2 [file mededu-v10-e54067-s002.pdf]

ChatGPT as a tool to help design educational materials in psychiatry

Thank you for taking the time to answer the following questionnaire.

This includes 6 clinical vignettes, some of which were generated by artificial intelligence (ChatGPT). We ask you to evaluate the clinical vignettes (for teaching-learning of psychiatry) according to very specific criteria.

The time to complete the questionnaire is estimated between 5 and 15 minutes.

There are 20 questions in this questionnaire.

Sociodemographic information

What is your level of training?

Please select an answer below
Please select only one of the following options:

- ◯ Resident physician in psychiatry
- ◯ Clinician-teacher / Psychiatrist

What is your number of years of clinical experience (including residency)?

Please select an answer below
Please select only one of the following options:

- ◯ 0 to 5 years
- ◯ 6 to 10 years
- ◯ More than 10 years

Vignette 1

Here is the first thumbnail. Please read it carefully.

Situation professionnelle authentique :

Julien, 22 ans, se présente à l'urgence psychiatrique. Il souligne avoir beaucoup de difficulté à se concentrer dans ses études et la réalisation de ses travaux scolaires depuis quelques semaines. Facilement distrait, il lui arrive d'être fréquemment dans la lune.

Si vous pensiez ...	Et qu'alors...	Votre hypothèse en est ...
**1- À une dépression majeure**	Monsieur soulève avoir une fatigue importante durant la journée et se réveille presqu'à toutes les nuits vers 3AM.	☐ Fortement affaiblie<br>☐ Affaiblie<br>☐ Inchangée<br>☐ Renforcée<br>☐ Fortement renforcée
**2- À un trouble d'anxiété généralisée**	Monsieur dit être convaincu d'avoir une maladie grave.	☐ Fortement affaiblie<br>☐ Affaiblie<br>☐ Inchangée<br>☐ Renforcée<br>☐ Fortement renforcée
**3- À un trouble déficitaire de l'attention**	Monsieur était premier de classe au primaire.	☐ Fortement affaiblie<br>☐ Affaiblie<br>☐ Inchangée<br>☐ Renforcée<br>☐ Fortement renforcée

Situation professionnelle authentique :

Julien, 22 ans, se présente à l'urgence psychiatrique. Il souligne avoir beaucoup de difficulté à se concentrer dans ses études et la réalisation de ses travaux scolaires depuis quelques semaines. Facilement distrait, il lui arrive d'être fréquemment dans la lune.

Si vous pensiez ...	Et qu'alors...	Votre hypothèse en est ...
**1- À une dépression majeure**	Monsieur soulève avoir une fatigue importante durant la journée et se réveille presqu'à toutes les nuits vers 3AM.	☐ Fortement affaiblie<br>☐ Affaiblie<br>☐ Inchangée<br>☐ Renforcée<br>☐ Fortement renforcée
**2- À un trouble d'anxiété généralisée**	Monsieur dit être convaincu d'avoir une maladie grave.	☐ Fortement affaiblie<br>☐ Affaiblie<br>☐ Inchangée<br>☐ Renforcée<br>☐ Fortement renforcée
**3- À un trouble déficitaire de l'attention**	Monsieur était premier de classe au primaire.	☐ Fortement affaiblie<br>☐ Affaiblie<br>☐ Inchangée<br>☐ Renforcée<br>☐ Fortement renforcée

Choose the appropriate answer for each item:

	Yes	Uncertain	Non
**The vignette describes a difficult situation, even for experts**	◯	◯	◯
**The vignette describes a situation appropriate for medical students**	◯	◯	◯
**The scenario is necessary to understand the question and to set the context**	◯	◯	◯
**The clinical presentation is typical**	◯	◯	◯
**The screenplay is well written**	◯	◯	◯
**The questions are developed using a key element approach (addition of an important clinical element)**	◯	◯	◯
**In your opinion, the options (proposed questions) are relevant**	◯	◯	◯
**The same option (e.g.: diagnosis) is not found in two consecutive questions**	◯	◯	◯
**The new information (2nd column) makes it possible to test the link between the new information and the option (1st column) in the context described**	◯	◯	◯
**Likert scale anchors (last column) are clearly defined and unambiguous**	◯	◯	◯
**Questions are expanded to distribute responses equally across all Likert scale values**	◯	◯	◯
**Questions are designed to provide a balance between low and high variability (between questions)**	◯	◯	◯
**In your opinion, was the thumbnail made from ChatGPT?**	◯	◯	◯

What do you think is the main diagnosis reflected by this clinical vignette? *

Please write your answer here:

What are the strengths and weaknesses of the vignette? *

Please write your answer here:

Vignette 2

Here is the second vignette. Please read it carefully.

Situation professionnelle authentique :

M. Dupont, âgé de 35 ans, est un vétéran de l'armée. Il présente des cauchemars récurrents, des flashbacks et une forte anxiété lorsqu'il entend des bruits similaires à ceux de tirs d'armes à feu.

Si vous pensiez ...	Et qu'alors...	Votre hypothèse en est ...
**1- À un trouble stress post-traumatique**	Monsieur mentionne éviter les situations qui lui rappelle les armes à feu	☐ Fortement affaiblie<br>☐ Affaiblie<br>☐ Inchangée<br>☐ Renforcée<br>☐ Fortement renforcée
**2- À un trouble dépressif caractérisé**	Monsieur dit être avoir de graves problèmes de concentration	☐ Fortement affaiblie<br>☐ Affaiblie<br>☐ Inchangée<br>☐ Renforcée<br>☐ Fortement renforcée
**3- À un trouble d'adaptation avec humeur anxieuse**	Monsieur mentionne que l'anxiété est aussi intense dans les autres sphères de sa vie	☐ Fortement affaiblie<br>☐ Affaiblie<br>☐ Inchangée<br>☐ Renforcée<br>☐ Fortement renforcée

Situation professionnelle authentique :

M. Dupont, âgé de 35 ans, est un vétéran de l'armée. Il présente des cauchemars récurrents, des flashbacks et une forte anxiété lorsqu'il entend des bruits similaires à ceux de tirs d'armes à feu.

Si vous pensiez ...	Et qu'alors...	Votre hypothèse en est ...
**1- À un trouble stress post-traumatique**	Monsieur mentionne éviter les situations qui lui rappelle les armes à feu	☐ Fortement affaiblie<br>☐ Affaiblie<br>☐ Inchangée<br>☐ Renforcée<br>☐ Fortement renforcée
**2- À un trouble dépressif caractérisé**	Monsieur dit être avoir de graves problèmes de concentration	☐ Fortement affaiblie<br>☐ Affaiblie<br>☐ Inchangée<br>☐ Renforcée<br>☐ Fortement renforcée
**3- À un trouble d'adaptation avec humeur anxieuse**	Monsieur mentionne que l'anxiété est aussi intense dans les autres sphères de sa vie	☐ Fortement affaiblie<br>☐ Affaiblie<br>☐ Inchangée<br>☐ Renforcée<br>☐ Fortement renforcée

*

Choose the appropriate answer for each item:

	Yes	Uncertain	Non
**The vignette describes a difficult situation, even for experts**	○	○	○
**The vignette describes a situation appropriate for medical students**	○	○	○
**The scenario is necessary to understand the question and to set the context**	○	○	○
**The clinical presentation is typical**	○	○	○
**The screenplay is well written**	○	○	○
**The questions are developed using a key element approach (addition of an important clinical element)**	○	○	○
**In your opinion, the options (proposed questions) are relevant**	○	○	○
**The same option (e.g.: diagnosis) is not found in two consecutive questions**	○	○	○
**The new information (2nd column) makes it possible to test the link between the new information and the option (1st column) in the context described**	○	○	○
**Likert scale anchors (last column) are clearly defined and unambiguous**	○	○	○
**Questions are expanded to distribute responses equally across all Likert scale values**	○	○	○
**Questions are designed to provide a balance between low and high variability (between questions)**	○	○	○
**In your opinion, was the thumbnail made from ChatGPT?**	○	○	○

What do you think is the main diagnosis reflected by this clinical vignette? *

Please write your answer here:

What are the strengths and weaknesses of the vignette? *

Please write your answer here:

Vignette 3

Here is the third vignette. Please read it carefully.

Situation professionnelle authentique :

Sophie est une femme âgée de 28 ans, est comptable. Elle passe la plupart de son temps libre à vérifier constamment les portes et les fenêtres de sa maison, craignant qu'elles ne soient pas correctement verrouillées. Elle se lave également les mains à répétition, souvent jusqu'à ce qu'elles soient rouges et irritées.

Si vous pensiez ...	Et qu'alors...	Votre hypothèse en est ...
**1- À un trouble d'anxiété généralisé**	Sophie mentionne que ses préoccupations sont seulement lorsqu'elle est chez elle	☐ Fortement affaiblie<br>☐ Affaiblie<br>☐ Inchangée<br>☐ Renforcée<br>☐ Fortement renforcée
**2- À un trouble de la personnalité obsessionnelle-compulsive**	Sophie rapporte être perfectionniste de nature	☐ Fortement affaiblie<br>☐ Affaiblie<br>☐ Inchangée<br>☐ Renforcée<br>☐ Fortement renforcée
**3- À un trouble obsessionnel-compulsif**	Sophie dit que ses symptômes interfèrent avec son fonctionnement quotidien	☐ Fortement affaiblie<br>☐ Affaiblie<br>☐ Inchangée<br>☐ Renforcée<br>☐ Fortement renforcée

Situation professionnelle authentique :

Sophie est une femme âgée de 28 ans, est comptable. Elle passe la plupart de son temps libre à vérifier constamment les portes et les fenêtres de sa maison, craignant qu'elles ne soient pas correctement verrouillées. Elle se lave également les mains à répétition, souvent jusqu'à ce qu'elles soient rouges et irritées.

Si vous pensiez ...	Et qu'alors...	Votre hypothèse en est ...
**1- À un trouble d'anxiété généralisé**	Sophie mentionne que ses préoccupations sont seulement lorsqu'elle est chez elle	☐ Fortement affaiblie<br>☐ Affaiblie<br>☐ Inchangée<br>☐ Renforcée<br>☐ Fortement renforcée
**2- À un trouble de la personnalité obsessionnelle-compulsive**	Sophie rapporte être perfectionniste de nature	☐ Fortement affaiblie<br>☐ Affaiblie<br>☐ Inchangée<br>☐ Renforcée<br>☐ Fortement renforcée
**3- À un trouble obsessionnel-compulsif**	Sophie dit que ses symptômes interfèrent avec son fonctionnement quotidien	☐ Fortement affaiblie<br>☐ Affaiblie<br>☐ Inchangée<br>☐ Renforcée<br>☐ Fortement renforcée

*

Choose the appropriate answer for each item:

	Yes	Uncertain	Non
**The vignette describes a difficult situation, even for experts**	○	○	○
**The vignette describes a situation appropriate for medical students**	○	○	○
**The scenario is necessary to understand the question and to set the context**	○	○	○
**The clinical presentation is typical**	○	○	○
**The screenplay is well written**	○	○	○
**The questions are developed using a key element approach (addition of an important clinical element)**	○	○	○
**In your opinion, the options (proposed questions) are relevant**	○	○	○
**The same option (e.g.: diagnosis) is not found in two consecutive questions**	○	○	○
**The new information (2nd column) makes it possible to test the link between the new information and the option (1st column) in the context described**	○	○	○
**Likert scale anchors (last column) are clearly defined and unambiguous**	○	○	○
**Questions are expanded to distribute responses equally across all Likert scale values**	○	○	○
**Questions are designed to provide a balance between low and high variability (between questions)**	○	○	○
**In your opinion, was the thumbnail made from ChatGPT?**	○	○	○

What do you think is the main diagnosis reflected by this clinical vignette? *

Please write your answer here:

What are the strengths and weaknesses of the vignette? *

Please write your answer here:

Vignette 4

Here is the fourth vignette. Please read it carefully.

Situation professionnelle authentique :

Marie a 34 ans. Elle consulte fréquemment les médecins pour des symptômes variés tels que des douleurs abdominales, des maux de tête et des éruptions cutanées. Malgré des examens approfondis, aucune cause médicale sous-jacente n'est trouvée. Elle a également été hospitalisée plusieurs fois pour des symptômes graves, mais aucun diagnostic médical concret n'a été établi.

Si vous pensiez ...	Et qu'alors...	Votre hypothèse en est ...
**1- À un trouble factice**	Marie mentionne travailler comme infirmière dans un hôpital communautaire	☐ Fortement affaiblie<br>☐ Affaiblie<br>☐ Inchangée<br>☐ Renforcée<br>☐ Fortement renforcée
**2- À un trouble à symptomatologie somatique**	Marie présente par moment une paralysie du membre supérieur droit	☐ Fortement affaiblie<br>☐ Affaiblie<br>☐ Inchangée<br>☐ Renforcée<br>☐ Fortement renforcée
**3- À de la simulation**	Marie mentionne vouloir obtenir un statut d'invalidité afin de ne plus avoir à travailler	☐ Fortement affaiblie<br>☐ Affaiblie<br>☐ Inchangée<br>☐ Renforcée<br>☐ Fortement renforcée

Situation professionnelle authentique :

Marie a 34 ans. Elle consulte fréquemment les médecins pour des symptômes variés tels que des douleurs abdominales, des maux de tête et des éruptions cutanées. Malgré des examens approfondis, aucune cause médicale sous-jacente n'est trouvée. Elle a également été hospitalisée plusieurs fois pour des symptômes graves, mais aucun diagnostic médical concret n'a été établi.

Si vous pensiez ...	Et qu'alors...	Votre hypothèse en est ...
**1- À un trouble factice**	Marie mentionne travailler comme infirmière dans un hôpital communautaire	☐ Fortement affaiblie<br>☐ Affaiblie<br>☐ Inchangée<br>☐ Renforcée<br>☐ Fortement renforcée
**2- À un trouble à symptomatologie somatique**	Marie présente par moment une paralysie du membre supérieur droit	☐ Fortement affaiblie<br>☐ Affaiblie<br>☐ Inchangée<br>☐ Renforcée<br>☐ Fortement renforcée
**3- À de la simulation**	Marie mentionne vouloir obtenir un statut d'invalidité afin de ne plus avoir à travailler	☐ Fortement affaiblie<br>☐ Affaiblie<br>☐ Inchangée<br>☐ Renforcée<br>☐ Fortement renforcée

*

Choose the appropriate answer for each item:

	Yes	Uncertain	Non
**The vignette describes a difficult situation, even for experts**	◯	◯	◯
**The vignette describes a situation appropriate for medical students**	◯	◯	◯
**The scenario is necessary to understand the question and to set the context**	◯	◯	◯
**The clinical presentation is typical**	◯	◯	◯
**The screenplay is well written**	◯	◯	◯
**The questions are developed using a key element approach (addition of an important clinical element)**	◯	◯	◯
**In your opinion, the options (proposed questions) are relevant**	◯	◯	◯
**The same option (e.g.: diagnosis) is not found in two consecutive questions**	◯	◯	◯
**The new information (2nd column) makes it possible to test the link between the new information and the option (1st column) in the context described**	◯	◯	◯
**Likert scale anchors (last column) are clearly defined and unambiguous**	◯	◯	◯
**Questions are expanded to distribute responses equally across all Likert scale values**	◯	◯	◯
**Questions are designed to provide a balance between low and high variability (between questions)**	◯	◯	◯
**In your opinion, was the thumbnail made from ChatGPT?**	◯	◯	◯

What do you think is the main diagnosis reflected by this clinical vignette? *

Please write your answer here:

What are the strengths and weaknesses of the vignette? *

Please write your answer here:

Vignette 5

Here is the fifth vignette. Please read it carefully.

Situation professionnelle authentique :

Vous êtes demandés à l'étage en consultation pour Monsieur Tremblay 55 ans pour agitation sévère. Vous apprenez qu'il prend du Lithium et du Citalopram (Celexa).

Si vous pensiez ...	Et qu'alors...	Votre hypothèse en est ...
**1- Un syndrome sérotoninergique**	Vous apprenez qu'il a reçu de l'Haldol hier soir.	☐ Fortement affaiblie<br>☐ Affaiblie<br>☐ Inchangée<br>☐ Renforcée<br>☐ Fortement renforcée
**2- Une intoxication au Lithium.**	Vous recevez une alerte au laboratoire pour une créatinine à 300 mmol/L.	☐ Fortement affaiblie<br>☐ Affaiblie<br>☐ Inchangée<br>☐ Renforcée<br>☐ Fortement renforcée
**3- Un délirium**	À l'examen physique vous avez une rigidité musculaire importante aux membres supérieurs et inférieurs.	☐ Fortement affaiblie<br>☐ Affaiblie<br>☐ Inchangée<br>☐ Renforcée<br>☐ Fortement renforcée

Situation professionnelle authentique :

Vous êtes demandés à l'étage en consultation pour Monsieur Tremblay 55 ans pour agitation sévère. Vous apprenez qu'il prend du Lithium et du Citalopram (Celexa).

Si vous pensiez ...	Et qu'alors...	Votre hypothèse en est ...
**1- Un syndrome sérotoninergique**	Vous apprenez qu'il a reçu de l'Haldol hier soir.	☐ Fortement affaiblie<br>☐ Affaiblie<br>☐ Inchangée<br>☐ Renforcée<br>☐ Fortement renforcée
**2- Une intoxication au Lithium.**	Vous recevez une alerte au laboratoire pour une créatinine à 300 mmol/L.	☐ Fortement affaiblie<br>☐ Affaiblie<br>☐ Inchangée<br>☐ Renforcée<br>☐ Fortement renforcée
**3- Un délirium**	À l'examen physique vous avez une rigidité musculaire importante aux membres supérieurs et inférieurs.	☐ Fortement affaiblie<br>☐ Affaiblie<br>☐ Inchangée<br>☐ Renforcée<br>☐ Fortement renforcée

*

Choose the appropriate answer for each item:

	Yes	Uncertain	Non
**The vignette describes a difficult situation, even for experts**	○	○	○
**The vignette describes a situation appropriate for medical students**	○	○	○
**The scenario is necessary to understand the question and to set the context**	○	○	○
**The clinical presentation is typical**	○	○	○
**The screenplay is well written**	○	○	○
**The questions are developed using a key element approach (addition of an important clinical element)**	○	○	○
**In your opinion, the options (proposed questions) are relevant**	○	○	○
**The same option (e.g.: diagnosis) is not found in two consecutive questions**	○	○	○
**The new information (2nd column) makes it possible to test the link between the new information and the option (1st column) in the context described**	○	○	○
**Likert scale anchors (last column) are clearly defined and unambiguous**	○	○	○
**Questions are expanded to distribute responses equally across all Likert scale values**	○	○	○
**Questions are designed to provide a balance between low and high variability (between questions)**	○	○	○
**In your opinion, was the thumbnail made from ChatGPT?**	○	○	○

What do you think is the main diagnosis reflected by this clinical vignette?

Please write your answer here:

What are the strengths and weaknesses of the vignette? *

Please write your answer here:

Vignette 6

Here is the last vignette. Please read it carefully.

Situation professionnelle authentique :

Julie, 26 ans, est amenée à l'urgence par les policiers. Elle est agitée, hurle et mentionne être pourchassée. Mme n'a pas d'antécédents médicaux et ne prend pas de médicament.

Si vous pensiez ...	Et qu'alors...	Votre hypothèse en est ...
**1- À une maladie bipolaire affective de type 1**	Des informations collatérales nous indiquent que Madame ne dort pas depuis 2 jours.	☐ Fortement affaiblie<br>☐ Affaiblie<br>☐ Inchangée<br>☐ Renforcée<br>☐ Fortement renforcée
**2- À un trouble de la personnalité limite**	Madame a tendance à consommer du cannabis, de la cocaïne et de l'héroïne de façon impulsive en présence de ses amis.	☐ Fortement affaiblie<br>☐ Affaiblie<br>☐ Inchangée<br>☐ Renforcée<br>☐ Fortement renforcée
**3- À une schizophrénie**	La sœur de Madame est atteinte de schizophrénie.	☐ Fortement affaiblie<br>☐ Affaiblie<br>☐ Inchangée<br>☐ Renforcée<br>☐ Fortement renforcée

Situation professionnelle authentique :

Julie, 26 ans, est amenée à l'urgence par les policiers. Elle est agitée, hurle et mentionne être pourchassée. Mme n'a pas d'antécédents médicaux et ne prend pas de médicament.

Si vous pensiez ...	Et qu'alors...	Votre hypothèse en est ...
**1- À une maladie bipolaire affective de type 1**	Des informations collatérales nous indiquent que Madame ne dort pas depuis 2 jours.	☐ Fortement affaiblie<br>☐ Affaiblie<br>☐ Inchangée<br>☐ Renforcée<br>☐ Fortement renforcée
**2- À un trouble de la personnalité limite**	Madame a tendance à consommer du cannabis, de la cocaïne et de l'héroïne de façon impulsive en présence de ses amis.	☐ Fortement affaiblie<br>☐ Affaiblie<br>☐ Inchangée<br>☐ Renforcée<br>☐ Fortement renforcée
**3- À une schizophrénie**	La sœur de Madame est atteinte de schizophrénie.	☐ Fortement affaiblie<br>☐ Affaiblie<br>☐ Inchangée<br>☐ Renforcée<br>☐ Fortement renforcée

*

Choose the appropriate answer for each item:

	Yes	Uncertain	Non
**The vignette describes a difficult situation, even for experts**	◯	◯	◯
**The vignette describes a situation appropriate for medical students**	◯	◯	◯
**The scenario is necessary to understand the question and to set the context**	◯	◯	◯
**The clinical presentation is typical**	◯	◯	◯
**The screenplay is well written**	◯	◯	◯
**The questions are developed using a key element approach (addition of an important clinical element)**	◯	◯	◯
**In your opinion, the options (proposed questions) are relevant**	◯	◯	◯
**The same option (e.g.: diagnosis) is not found in two consecutive questions**	◯	◯	◯
**The new information (2nd column) makes it possible to test the link between the new information and the option (1st column) in the context described**	◯	◯	◯
**Likert scale anchors (last column) are clearly defined and unambiguous**	◯	◯	◯
**Questions are expanded to distribute responses equally across all Likert scale values**	◯	◯	◯
**Questions are designed to provide a balance between low and high variability (between questions)**	◯	◯	◯
**In your opinion, was the thumbnail made from ChatGPT?**	◯	◯	◯

What do you think is the main diagnosis reflected by this clinical vignette?

Please write your answer here:

What are the strengths and weaknesses of the vignette? *

Please write your answer here:

Thank you very much for your time and for completing the questionnaire!

If you have questions about the research project or if you wish to withdraw from the research project, you can contact the researcher responsible for this research project at the following coordinates: Dr. Alexandre Hudon, resident physician in psychiatry and project coordinator (alexandre.hudon.1@umontreal.ca , 514-995-4842).

10/21/2023 – 4:41 p.m.
Send your questionnaire.
Thank you for completing this questionnaire.

SCT translated from French to English

Vignette 1

Authentic professional situation: Julien, 22 years old, presents himself to the psychiatric emergency room. He emphasizes that he has had great difficulty concentrating in his studies and completing his school work for several weeks. Easily distracted, he often finds himself in the mood.		
If you thought ...	And then...	Your hypothesis is ...
1- Major depression	Mr. reports having significant fatigue during the day and wakes up almost every night around 3AM.	☐ Significantly weakened<br>☐ Weakened<br>☐ Unchanged<br>☐ Reinforced<br>☐ Strongly reinforced
2- Generalized anxiety disorder	The gentleman says he is convinced that he has a serious illness.	☐ Significantly weakened<br>☐ Weakened<br>☐ Unchanged<br>☐ Reinforced<br>☐ Strongly reinforced
3- Attention deficit disorder	Mr. was at the top of his class in primary school.	☐ Significantly weakened<br>☐ Weakened<br>☐ Unchanged<br>☐ Reinforced<br>☐ Strongly reinforced

Vignette 2

Authentic professional situation: Mr. Dupont, 35 years old, is an army veteran. He experiences recurring nightmares, flashbacks, and severe anxiety when hearing noises similar to gunshots.		
If you thought ...	And then...	Your hypothesis is ...
1- Post-traumatic stress disorder	Mr. mentions avoiding situations that remind him of guns	☐ Significantly weakened<br>☐ Weakened<br>☐ Unchanged<br>☐ Reinforced<br>☐ Strongly reinforced
2- A characterized depressive disorder	Gentleman says he has serious concentration problems	☐ Significantly weakened<br>☐ Weakened<br>☐ Unchanged<br>☐ Reinforced<br>☐ Strongly reinforced
3- An adjustment disorder with anxious mood	Monsieur mentions that the anxiety is also intense in other areas of his life	☐ Significantly weakened<br>☐ Weakened<br>☐ Unchanged<br>☐ Reinforced<br>☐ Strongly reinforced

Vignette 3

Authentic professional situation: Sophie is a 28 year old woman and is an accountant. She spends most of her free time constantly checking the doors and windows of her house, worrying that they are not properly locked. She also washes her hands repeatedly, often until they are red and irritated.		
If you thought ...	And then...	Your hypothesis is...
1- Generalized anxiety disorder	Sophie mentions that her concerns are only when she is at home	☐ Significantly weakened<br>☐ Weakened<br>☐ Unchanged<br>☐ Reinforced<br>☐ Strongly reinforced
2- Obsessive-compulsive personality disorder	Sophie reports being a perfectionist by nature	☐ Significantly weakened<br>☐ Weakened<br>☐ Unchanged<br>☐ Reinforced<br>☐ Strongly reinforced
3- Obsessive-compulsive disorder	Sophie says her symptoms interfere with her daily functioning	☐ Significantly weakened<br>☐ Weakened<br>☐ Unchanged<br>☐ Reinforced<br>☐ Strongly reinforced

Vignette 4

Authentic professional situation:

Mary is 34 years old. She frequently visits doctors for various symptoms such as abdominal pain, headaches and rashes. Despite extensive examinations, no underlying medical cause was found. She was also hospitalized several times for serious symptoms, but no concrete medical diagnosis was made.

If you thought ...	And then...	Your hypothesis is...
1- Factitious disorder	Marie mentions working as a nurse in a community hospital	☐ Significantly weakened<br>☐ Weakened<br>☐ Unchanged<br>☐ Reinforced<br>☐ Strongly reinforced
2- A disorder with somatic symptoms	Marie sometimes presents with paralysis of the right upper limb.	☐ Significantly weakened<br>☐ Weakened<br>☐ Unchanged<br>☐ Reinforced<br>☐ Strongly reinforced
3- To simulation	Marie mentions wanting to obtain disability status so that she no longer has to work	☐ Significantly weakened<br>☐ Weakened<br>☐ Unchanged<br>☐ Reinforced<br>☐ Strongly reinforced

Vignette 5

<

Authentic professional situation:

You are requested upstairs for consultation for Mr. Tremblay, 55 years old, for severe agitation. You learn that he takes Lithium and Citalopram (Celexa).

If you thought ...	And then...	Your hypothesis is...
1- Serotonin syndrome	You learn that he received Haldol last night.	☐ Significantly weakened<br>☐ Weakened<br>☐ Unchanged<br>☐ Reinforced<br>☐ Strongly reinforced
2- Lithium poisoning.	You receive an alert at the laboratory for a creatinine of 300 mmol/L.	☐ Significantly weakened<br>☐ Weakened<br>☐ Unchanged<br>☐ Reinforced<br>☐ Strongly reinforced
3- A delirium	On physical examination you have significant muscle rigidity in the upper and lower limbs.	☐ Significantly weakened<br>☐ Weakened<br>☐ Unchanged<br>☐ Reinforced<br>☐ Strongly reinforced

Vignette 6

Authentic professional situation:

Julie, 26, is brought to the emergency room by the police. She is agitated, screams and mentions being chased. Ms. has no medical history and is not taking medication.

If you thought...	And then...	Your hypothesis is ...
1- Type 1 affective bipolar illness	Collateral information tells us that Madame has not slept for 2 days.	☐ Significantly weakened<br>☐ Weakened<br>☐ Unchanged<br>☐ Reinforced<br>☐ Strongly reinforced
2- Borderline personality disorder	Madame tends to use cannabis, cocaine and heroin impulsively in the presence of her friends.	☐ Significantly weakened<br>☐ Weakened<br>☐ Unchanged<br>☐ Reinforced<br>☐ Strongly reinforced
3- To schizophrenia	Madame's sister suffers from schizophrenia.	☐ Significantly weakened<br>☐ Weakened<br>☐ Unchanged<br>☐ Reinforced<br>☐ Strongly reinforced
