# Supplementary material for: Using ChatGPT in Psychiatry to Design Script Concordance Tests in Undergraduate Medical Education: Mixed Methods Study
Source: JMIR Med Educ. 2024 Apr 4;10:e54067. doi: 10.2196/54067 (PMC11007379; doi:10.2196/54067)
Supplement: Multimedia Appendix 3 [file mededu-v10-e54067-s003.docx]

**Multimedia Appendix 3.** Responses for every component of the SCT evaluations for the six SCTs as per the category of respondents.

| **Questions**  **SCT 1** | **SCT 1**  **Medical resident**  **(N = 45)** | **SCT 1**  **Teaching physician (less than 5 years)**  **(N = 2)** | **SCT 1**  **Teaching physician (6-10 years)**  **(N = 16)** | **SCT 1**  **Teaching physician (more than 10 years)**  **(N = 39)** | **SCT1 Pooled**  **(N = 102)** |
| --- | --- | --- | --- | --- | --- |
| **S1 Yes(N,%)** | 12, 26.67 | 1, 50.00 | 4, 25.00 | 10, 25.64 | 27, 26.47 |
| **S2 Yes(N,%)** | 39, 86.67 | 2, 100.00 | 14, 87.50 | 29, 75.36 | 84, 82.35 |
| **S3 Yes(N,%)** | 41, 91.11 | 2, 100.00 | 14, 87.50 | 29, 75.36 | 86, 84.31 |
| **S4 Yes(N,%)** | 27, 60.00 | 2, 100.00 | 14, 87.50 | 29, 75.36 | 72, 70.59 |
| **S5 Yes(N,%)** | 33, 73.33 | 2, 100.00 | 14, 87.50 | 31, 79.49 | 80, 78.43 |
| **Q1 Yes(N,%)** | 33, 73.33 | 2, 100.00 | 14, 87.50 | 31, 79.49 | 80, 78.43 |
| **Q2 Yes(N,%)** | 33, 73.33 | 2, 100.00 | 14, 87.50 | 31, 79.49 | 80, 78.43 |
| **Q3 Yes(N,%)** | 31, 68.89 | 2, 100.00 | 12, 75.00 | 27, 69.23 | 72, 70.59 |
| **Q4 Yes(N,%)** | 33, 73.33 | 2, 100.00 | 12, 75.00 | 31, 79.48 | 78, 76.47 |
| **Q5 Yes(N,%)** | 37, 82.22 | 2, 100.00 | 14, 87.50 | 29, 74.36 | 82, 80.39 |
| **Q6 Yes(N,%)** | 22, 48.89 | 2, 100.00 | 14, 87.50 | 31, 79.49 | 69, 67.65 |
| **Q7 Yes(N,%)** | 18, 40.00 | 2, 100.00 | 12, 75.00 | 20, 51.28 | 52, 50.98 |
| **Is it a ChatGPT generated scenario? Correct answers (N, %)** | 25, 44.44 | 1, 50.00 | 6, 37.50 | 18, 53.85 | 50, 49.02 |
| **Questions**  **SCT 2** | **SCT 2^a^**  **Medical resident** | **SCT 2 ^a^**  **Teaching physician (less than 5 years)** | **SCT 2 ^a^**  **Teaching physician (6-10 years)** | **SCT 2 ^a^**  **Teaching physician (more than 10 years)** | **SCT 2 ^a^ Pooled** |
| **S1 Yes(N,%)** | 11, 24.44 | 1, 50.00 | 4, 25.00 | 9, 23.10 | 25, 24.51 |
| **S2 Yes(N,%)** | 33, 73.33 | 1, 50.00 | 14, 87.50 | 29, 74.36 | 77, 75.49 |
| **S3 Yes(N,%)** | 31, 68.89 | 2, 100.00 | 16, 100.00 | 27, 69.23 | 76, 74.51 |
| **S4 Yes(N,%)** | 35, 77.78 | 2, 100.00 | 16, 100.00 | 31, 79.49 | 84, 82.35 |
| **S5 Yes(N,%)** | 35, 77.78 | 2, 100.00 | 16, 100.00 | 29, 74.36 | 82, 80.39 |
| **Q1 Yes(N,%)** | 37, 82.22 | 2, 100.00 | 10, 62.50 | 23, 58.97 | 72, 70.59 |
| **Q2 Yes(N,%)** | 29, 64.44 | 2, 100.00 | 12, 75.00 | 21, 53.85 | 64, 62.74 |
| **Q3 Yes(N,%)** | 27, 60.00 | 2, 100.00 | 12, 75.00 | 27, 69.23 | 68, 66.67 |
| **Q4 Yes(N,%)** | 35, 77.78 | 2, 100.00 | 10, 62.50 | 23, 58.97 | 70, 68.63 |
| **Q5 Yes(N,%)** | 35, 77.78 | 2, 100.00 | 16, 100.00 | 29, 74.36 | 82, 80.39 |
| **Q6 Yes(N,%)** | 23, 51.11 | 2, 100.00 | 14, 87.50 | 31, 79.49 | 70, 68.63 |
| **Q7 Yes(N,%)** | 23, 51.11 | 2, 100.00 | 12, 75.00 | 20, 51.28 | 57, 55.88 |
| **Is it ChatGPT Correct answers (N, %)** | 22, 48.89 | 0, 0.00 | 6, 37.50 | 18, 46.15 | 46, 45.10 |
| **Questions**  **SCT 3** | **SCT 3 ^a^**  **Medical resident** | **SCT 3 ^a^**  **Teaching physician (less than 5 years)** | **SCT 3 ^a^**  **Teaching physician (6-10 years)** | **SCT 3 ^a^**  **Teaching physician (more than 10 years)** | **SCT 3 ^a^ Pooled** |
| **S1 Yes(N,%)** | 14, 41.11 | 1, 50.00 | 4, 25.00 | 12, 30.70 | 31, 30.39 |
| **S2 Yes(N,%)** | 29, 64.44 | 2, 100.00 | 14, 87.50 | 29, 74.36 | 74, 72.55 |
| **S3 Yes(N,%)** | 35, 77.78 | 2, 100.00 | 14, 87.50 | 27, 69.23 | 78, 76.47 |
| **S4 Yes(N,%)** | 29, 64.44 | 2, 100.00 | 14, 87.50 | 31, 79.49 | 76, 74.51 |
| **S5 Yes(N,%)** | 31, 68.89 | 2, 100.00 | 14, 87.50 | 31, 79.49 | 78, 76.47 |
| **Q1 Yes(N,%)** | 29, 64.44 | 2, 100.00 | 14, 87.50 | 25, 64.10 | 70, 68.63 |
| **Q2 Yes(N,%)** | 27, 60.00 | 2, 100.00 | 14, 87.50 | 27, 69.23 | 70, 68.63 |
| **Q3 Yes(N,%)** | 31, 68.89 | 2, 100.00 | 12, 75.00 | 21, 53.85 | 66, 64.71 |
| **Q4 Yes(N,%)** | 31, 68.89 | 2, 100.00 | 12, 75.00 | 23, 58.97 | 68, 66.67 |
| **Q5 Yes(N,%)** | 27, 60.00 | 2, 100.00 | 16, 100.00 | 23, 58.97 | 68, 66.67 |
| **Q6 Yes(N,%)** | 25, 55.56 | 2, 100.00 | 12, 75.00 | 27, 69.23 | 66, 64.71 |
| **Q7 Yes(N,%)** | 23, 51.11 | 2, 100.00 | 14, 87.50 | 31, 79.49 | 70, 68.63 |
| **Is it ChatGPT Correct answers (N, %)** | 16, 35.56 | 0, 0.00 | 4, 25.00 | 16, 41.03 | 36, 35.29 |
| **Questions**  **SCT 4** | **SCT 4 ^a^**  **Medical resident** | **SCT 4 ^a^**  **Teaching physician (less than 5 years)** | **SCT 4 ^a^**  **Teaching physician (6-10 years)** | **SCT 4 ^a^**  **Teaching physician (more than 10 years)** | **SCT 4 ^a^ Pooled** |
| **S1 Yes(N,%)** | 22, 48.89 | 1, 50.00 | 6, 37.50 | 18, 46.15 | 47, 46.08 |
| **S2 Yes(N,%)** | 25, 55.56 | 2, 100.00 | 10, 62.50 | 31, 79.49 | 68, 66.67 |
| **S3 Yes(N,%)** | 31, 68.89 | 2, 100.00 | 14, 87.50 | 29, 74.36 | 74, 72.55 |
| **S4 Yes(N,%)** | 29, 64.44 | 2, 100.00 | 10, 62.50 | 25, 64.10 | 66, 64.71 |
| **S5 Yes(N,%)** | 31, 68.89 | 1, 50.00 | 14, 87.50 | 27, 69.23 | 73, 71.57 |
| **Q1 Yes(N,%)** | 31, 68.89 | 2, 100.00 | 14, 87.50 | 27, 69.23 | 74, 72.55 |
| **Q2 Yes(N,%)** | 29, 64.44 | 2, 100.00 | 14, 87.50 | 31, 79.49 | 76, 74.51 |
| **Q3 Yes(N,%)** | 25, 55.56 | 2, 100.00 | 14, 87.50 | 25, 64.10 | 66, 64.71 |
| **Q4 Yes(N,%)** | 25, 55.56 | 2, 100.00 | 14, 87.50 | 27, 69.23 | 70, 68.63 |
| **Q5 Yes(N,%)** | 25, 55.56 | 2, 100.00 | 14, 87.50 | 29, 74.36 | 70, 68.63 |
| **Q6 Yes(N,%)** | 21, 46.67 | 2, 100.00 | 14, 87.50 | 29, 74.36 | 66, 64.71 |
| **Q7 Yes(N,%)** | 21, 46.67 | 1, 50.00 | 14, 87.50 | 27, 69.23 | 63, 61.76 |
| **Is it ChatGPT Correct answers (N, %)** | 19, 42.22 | 1, 50.00 | 6, 37.50 | 12, 30.77 | 38, 37.25 |
| **Questions**  **SCT 5** | **SCT 5 ^a^**  **Medical resident** | **SCT 5 ^a^**  **Teaching physician (less than 5 years)** | **SCT 5 ^a^**  **Teaching physician (6-10 years)** | **SCT 5 ^a^**  **Teaching physician (more than 10 years)** | **SCT 5 ^a^ Pooled** |
| **S1 Yes(N,%)** | 22, 48.89 | 1, 50.00 | 6, 37.50 | 18, 46.15 | 47, 46.08 |
| **S2 Yes(N,%)** | 23, 51.11 | 2, 100.00 | 10, 62.50 | 27, 69.23 | 62, 60.78 |
| **S3 Yes(N,%)** | 29, 64.44 | 1, 50.00 | 14, 87.50 | 31, 79.49 | 75, 73.53 |
| **S4 Yes(N,%)** | 23, 51.11 | 2, 100.00 | 14, 87.50 | 29, 74.36 | 68, 66.67 |
| **S5 Yes(N,%)** | 31, 68.89 | 2, 100.00 | 10, 62.50 | 25, 64.10 | 68, 66.67 |
| **Q1 Yes(N,%)** | 29, 64.44 | 2, 100.00 | 12, 75.00 | 25, 64.10 | 68, 66.67 |
| **Q2 Yes(N,%)** | 29, 64.44 | 2, 100.00 | 12, 75.00 | 25, 64.10 | 68, 66.67 |
| **Q3 Yes(N,%)** | 29, 64.44 | 2, 100.00 | 14, 87.50 | 29, 74.36 | 74, 72.55 |
| **Q4 Yes(N,%)** | 29, 64.44 | 2, 100.00 | 12, 75.00 | 31, 79.49 | 74, 72.55 |
| **Q5 Yes(N,%)** | 29, 64.44 | 2, 100.00 | 14, 87.50 | 27, 69.23 | 72, 70.59 |
| **Q6 Yes(N,%)** | 23, 51.11 | 2, 100.00 | 14, 87.50 | 29, 74.36 | 68, 66.67 |
| **Q7 Yes(N,%)** | 23, 51.11 | 2, 100.00 | 14, 87.50 | 29, 74.36 | 68, 66.67 |
| **Is it ChatGPT Correct answers (N, %)** | 21, 53.33 | 2, 100.00 | 8, 50.00 | 23, 58.97 | 54, 52.94 |
| **Questions**  **SCT 6** | **SCT 6**  **Medical resident** | **SCT 6**  **Teaching physician (less than 5 years)** | **SCT 6**  **Teaching physician (6-10 years)** | **SCT 6**  **Teaching physician (more than 10 years)** | **SCT 6  Pooled** |
| **S1 Yes(N,%)** | 22, 48.89 | 1, 50.00 | 6, 37.50 | 14, 35.90 | 43, 42.57 |
| **S2 Yes(N,%)** | 25, 55,56 | 2, 100.00 | 12, 75.00 | 25, 64.10 | 64, 62.75 |
| **S3 Yes(N,%)** | 29, 64.44 | 2, 100.00 | 14, 87.50 | 31, 79.49 | 76, 74.51 |
| **S4 Yes(N,%)** | 29, 64.44 | 2, 100.00 | 12, 75.00 | 31, 79.49 | 72, 70.59 |
| **S5 Yes(N,%)** | 29, 64.44 | 2, 100.00 | 14, 87.50 | 27, 69.23 | 72, 70.59 |
| **Q1 Yes(N,%)** | 29, 64.44 | 2, 100.00 | 14, 87.50 | 27, 69.23 | 72, 70.59 |
| **Q2 Yes(N,%)** | 27, 60.00 | 2, 100.00 | 12, 75.00 | 25, 64.10 | 66, 64.71 |
| **Q3 Yes(N,%)** | 27, 60.00 | 2, 100.00 | 14, 87.50 | 27, 69.23 | 70, 68.63 |
| **Q4 Yes(N,%)** | 23, 51.11 | 2, 100.00 | 12, 75.00 | 27, 69.23 | 64, 62.75 |
| **Q5 Yes(N,%)** | 27, 60.00 | 2, 100.00 | 14, 87.50 | 27, 69.23 | 70, 68.63 |
| **Q6 Yes(N,%)** | 20, 44.44 | 2, 100.00 | 12, 75.00 | 29, 74.36 | 63, 61.76 |
| **Q7 Yes(N,%)** | 18, 40.00 | 2, 100.00 | 12, 75.00 | 29, 74.36 | 61, 59.80 |
| **Is it ChatGPT Correct answers (N, %)** | 21, 53.33 | 1, 50.00 | 8, 50.00 | 18, 46.15 | 48, 47.06 |

**^a^** SCT created by ChatGPT.
